# Effect of Peak Tracking Methods on FBG Calibration Derived by Factorial Design of Experiment

**DOI:** 10.3390/s21186169

**Published:** 2021-09-14

**Authors:** Nazila Safari Yazd, Jennifer Kawakami, Alireza Izaddoost, Patrice Mégret

**Affiliations:** 1 Electromagnetism and Telecommunication Department, University of Mons, 7000 Mons, Belgium; patrice.megret@umons.ac.be; 2 Department of Computer Science, California State University-Dominguez Hills (CSUDH), Carson, CA 90747, USA; jkawakami1@toromail.csudh.edu (J.K.); aizaddoost@csudh.edu (A.I.)

**Keywords:** fiber Bragg grating, design of experiment, multiparameter sensing, peak tracking

## Abstract

We present a calibration procedure for a humidity sensor made of a fiber Bragg grating covered by a polyimide layer. FBGs being intrinsically sensitive to temperature and strain, the calibration should tackle three variables, and, therefore, consists of a three-variable, two-level factorial design tailored to assess the three main sensitivities, as well as the five cross-sensitivities. FBG sensing information is encoded in the reflection spectrum from which the Bragg wavelength should be extracted. We tested six classical peak tracking methods on the results of the factorial design of the experiment applied to a homemade FBG humidity sensor. We used Python programming to compute, from the raw spectral data with six typical peak search algorithms, the temperature, strain and humidity sensitivities, as well as the cross-sensitivities, and showed that results are consistent for all algorithms, provided that the points selected to make the computation are correctly chosen. The best results for this particular sensor are obtained with a 3 dB threshold, whatever the peak search method used, and allow to compute the effective humidity sensitivity taking into account the combined effect of temperature and strain. The calibration procedure presented here is nevertheless generic and can thus be adapted to other sensors.

## 1. Introduction

Fiber Bragg grating (FBG) is a periodic and permanent modulation of the refractive index of the optical fiber core, which is achieved by exposing the optical fiber core to the interference pattern of ultraviolet light [1,2,3,4,5].

When light propagates through an FBG, a specific wavelength, referred to as the Bragg wavelength $\lambda_{B}$, is reflected in phase by each grating plane, whereas the remaining wavelengths pass through it. The Bragg wavelength depends on the effective refractive index of the core and the grating period. Both parameters are inherently temperature and axial strain-dependent. Therefore, any modification in temperature or strain causes the Bragg wavelength to change, and this property is the basis for an FBG sensor: by monitoring the Bragg wavelength, strain or temperature can be monitored. This is achieved by an FBG interrogator system that exists in various types, such as scanning filter setup [6,7], tunable laser setup [8,9] and spectroscopic setup [10]. Whatever the detection system used, the output is the FBG spectrum versus the wavelength from which the Bragg peak is extracted by standard algorithms (carried out in MATLAB or LabVIEW programming languages in most commercial equipment). If a sensitive layer to external stimuli (such as humidity) is added around the FBG, this stimuli can be measured by following the related peak shift and by calibrating the active layer. FBG-based sensors can therefore be adapted to sense various physical quantities and have numerous properties. FBG-based sensors are lightweight, small-sized, and passive. They are immune to electromagnetic interference as well. The FBGs are resistant to corrosion and highly sensitive. They respond fast and are capable of remote operation. In addition, FBGs have the potential for quasi-distributed sensing. They have been used to monitor temperature, humidity, strain, external refractive index, and bending [11,12,13,14,15,16,17,18,19]. Moreover, FBG-based sensors can be operated in harsh environments with severe physical/chemical conditions such as very high temperature, and high pressure [20,21,22,23,24,25,26].

From the grating physical principle, it is clear that any FBG is always sensitive to temperature and strain. If a polyimide sensitive layer is added to transform the FBG into a humidity sensor, the resulting sensor is sensitive to three parameters (temperature, strain and humidity). Therefore, calibration against the three parameters should be carried out, such that humidity can be retrieved as soon as temperature and strain are known. These two quantities can be measured by other types of sensors, as well as by FBGs without sensitive layer, one being packaged to be strain-independent.

The classical experimental approach to measure the effect of multi-factors (variables) on the output of a sensor is to study each variable separately. This approach is time consuming and does not allow to estimate the interactions between the factors [27,28]. For example, to study the effect of three factors on a phenomenon, if each factor, for instance, has seven levels (points of experiment or runs), the experimenter would have to carry out $7^{3}=343$ experiments or trials, which consumes time and is prone to error. The only way to reduce the experimental time is to decrease the number of levels per factor.

In contrast, a design of experiment (DoE) is a better strategy and more efficient for experimenting with multiple factors. In particular, a factorial design (FD) consists of modifying all the variables in each trial so that (1) the total number of experiments is reduced, and (2) the interactions between factors can be detected [27,28,29]. The simplest factorial design is a two-level design for which each factor is tested at only two levels: the low level (minimum value of the variable, or −1) and the high level (maximum value of the variable, or +1) in the interest range of the measurement.

In 2019 [12], we reported a two-level FD for three factors (temperature, strain and humidity) for FBGs sensors. With only eight trials, i.e., $2^{3}$, the number of combinations of two levels for three variables, the three main sensitivities and five cross-interactions of 2×2 and 3×3 were computed from the Bragg wavelength shifts measured by an FBG interrogator system that assumes that the Bragg wavelength is the maximal value of the FBG reflection spectrum.

In any measurement system, an algorithm is used to transform the spectrum data into the peak wavelength of the Bragg grating. It is clear that this algorithm influences the output of the measurement system; therefore, comparing the different peak detection algorithms is worthwhile, especially for multiparameter sensing applying DoE.

This work explains the calibration procedure of an FBG humidity sensor and shows the effect of the peak algorithm used on the sensitivity and cross-sensitivity estimations of a DoE of three parameters (temperature, strain and humidity) using a polyimide-coated FBG as a humidity sensor. In particular, six classical peak search algorithms have been analyzed and compared: maximum, centroid, *X*-dB bandwidth, Gaussian fit, polynomial quadratic fit, and cubic-spline fit.

## 2. Sensor Fabrication

To fabricate the humidity sensor, we used single-mode standard optical Draka Bendbright fiber, and prior to the grating inscription, the optical fiber was hydrogenated. The Bragg grating is inscribed using an interferometric setup that contains a Lloyd mirror as a wavefront splitter and a ten-fold beam expander. In addition, an adjustable diaphragm was used to fabricate a 4 mm grating length. The laser system that was used in this inscription setup consists of a fiber laser from Azur Light System with an external cavity frequency doubler from Sirah that emits at 244 nm. The grating was annealed for one day at 100 ${}^{{}^{\circ}}C$ to remove the residual hydrogen in the fiber and stabilize the grating’s properties.

**Figure 1:**
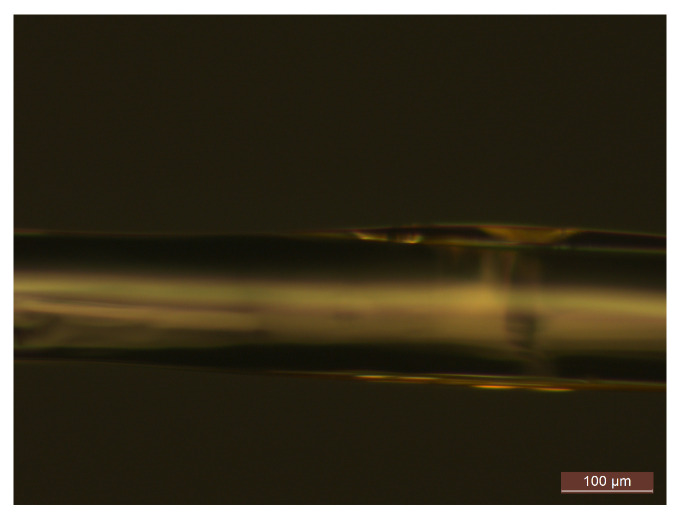
Microscope image of the PI-coated FBG that is deposited by hand on bare FBG in the lab.

Bare FBGs are not sensitive to humidity; therefore, for humidity sensing, we coated the FBG with an additional layer of polyimide (PI2525), which is known to be a hygroscopic material [30]. Figure 1 shows the PI layer coated on a bare FBG deposited by hand at the lab and leading to a polyimide thickness around 25 μm. PI possesses the water absorption property. When the coated grating with PI is in contact with humidity, the PI induces strain by swelling due to the absorption of the water molecules. There is a linear relation between the humidity and the volume expansion of this material [13]. This, in turn, induces strain that changes the grating period Λ and, consequently, the Bragg shift [13,31] is linearly proportional to humidity.

It is well-known from FBG theory [2] that the Bragg wavelength $\lambda_{B}$ for a first order diffraction grating is given by:

(1) $$\lambda_{B}=2n_{\mathrm{eff}}\Lambda$$

where $n_{\mathrm{eff}}$ is the effective refractive index of the guided mode in the optical fiber core, and Λ is the grating period, both temperature and strain-dependent [2]. Variations of temperature (*T*), strain (ε) and humidity (*H*) will therefore affect the Bragg wavelength of the PI-coated FBG, according to the generic expression:

(2) $$\lambda_{B}=f\left(T,H,\varepsilon \right)$$

The goal of the calibration is to estimate the function *f* from a number of measurements carefully chosen in the experimental space of the three variables *T*, ε and *H*.

## 3. Factorial Design

The most general surface response for a three-variable system is:

(3) $$y=f(x_{1},x_{2},x_{3})$$

where *f* is the unknown function to be estimated from calibration measurements. However, this equation is too general to be manageable. One way to tackle this problem is to make a Taylor series expansion:

(4) $$y=a_{0}+\sum_{i=1}^{3}a_{i}x_{i}+\sum_{i=1}^{3}\sum_{j=1}^{3}a_{ij}x_{i}x_{j}++\sum_{i=1}^{3}\sum_{j=1}^{3}\sum_{k=1}^{3}a_{ijk}x_{i}x_{j}x_{k}+\cdots$$

where *y* is the response, $x_{i}$ represents the level of factor *i*, $a_{0}$ (1 coefficient), $a_{i}$ (3 coefficients), $a_{ij}$ (9 coefficients of which 6 are independent) and $a_{ijk}$ (27 coefficients of which 10 are independent) are the coefficients of the polynomial representing the surface response:

(5) $$a_{0}=f\left(0,0,0\right),\;a_{i}={\left.\frac{\partial f}{\partial x_{i}}\right|}_{\left(0,0,0\right)},\;a_{ij}=\frac{1}{2}{\left.\frac{\partial^{2}f}{\partial x_{i}\partial x_{j}}\right|}_{\left(0,0,0\right)},\;a_{ijk}=\frac{1}{3!}{\left.\frac{\partial^{3}f}{\partial x_{i}\partial x_{j}\partial x_{k}}\right|}_{\left(0,0,0\right)}$$

In this study dedicated to humidity sensing with FBG, the ranges of temperature and strain (see Section 4) are small enough to consider a first-order model with interaction, i.e., we neglect all terms with powers higher than 1 in any variables $x_{i}$. Therefore, Equation (4) becomes:

(6) $$y=a_{0}+\sum_{i=1}^{n}a_{i}x_{i}+\sum_{i=1}^{n}\sum_{j\neq i}^{n}a_{ij}x_{i}x_{j}+a_{123}x_{1}x_{2}x_{3}$$

and from the symmetry of the partial derivatives versus the subscripts, $a_{ij}=a_{ji}$, so that (6) becomes:

(7) $$y=a_{0}+\sum_{i=1}^{n}a_{i}x_{i}+\sum_{i=1}^{n}\sum_{j=i+1}^{n}a_{ij}x_{i}x_{j}+a_{123}x_{1}x_{2}x_{3}$$

with $a_{ij}={\left.\partial^{2}f/\partial x_{i}\partial x_{j}\right|}_{\left(0,0,0\right)},i<j$.

With a first-order model with interaction, (1) if we fix two variables out of three, *y* varies linearly with the third variable, and (2) the slope for $x_{i}$ depends on $x_{j}$ and $x_{k}$. In terms of sensing, it means that the effective humidity sensitivity depends on the temperature and the strain.

**Figure 2:**
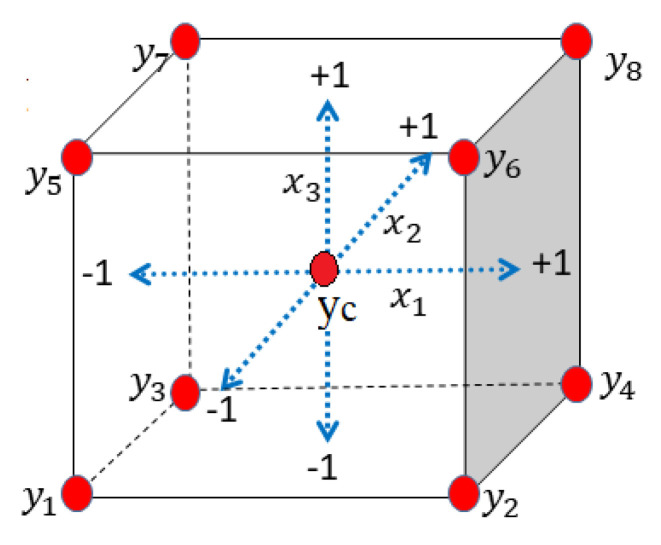
Two-level factorial design for three variables of temperature, humidity, and strain as $X_{t}$, $X_{h}$, and $X_{\varepsilon }$, respectively. The red points (vertices of cube) are the measurement points.

In DoE, it is useful to normalize the variables between −1 and +1, with −1 the minimum level and +1 the maximum level. For a two-level, three-variable system $x_{1}=X_{t}$, $x_{2}=X_{h}$ and $x_{3}=X_{\varepsilon }$ for temperature, humidity and strain, respectively, the trials are placed at eight vertexes ($y_{1}$ to $y_{8}$) of a cube, as shown in Figure 2. Moreover, a control point ($y_{c}$) is placed at the center of the cube and is used to validate the first-order model with interaction.

With these normalized variables, Equation (7) simply becomes:

(8) $$\lambda_{B}=A_{0}+A_{t}X_{t}+A_{h}X_{h}+A_{\varepsilon }X_{\varepsilon }+A_{th}X_{t}X_{h}+A_{t\varepsilon }X_{t}X_{\varepsilon }+A_{h\varepsilon }X_{h}X_{\varepsilon }+A_{th\varepsilon }X_{t}X_{h}X_{\varepsilon }$$

or in denormalized form:

(9) $$\lambda_{B}=S_{0}+S_{t}\Delta T+S_{h}\Delta H+S_{\varepsilon }\Delta \varepsilon +S_{th}\Delta T\Delta H+S_{t\varepsilon }\Delta T\Delta \varepsilon +S_{h\varepsilon }\Delta H\Delta \varepsilon +S_{th\varepsilon }\Delta T\Delta H\Delta \varepsilon$$

where the coefficients $A_{\mu }$ are linked to the coefficients $S_{\nu }$ by simple relationships [27].

In summary, relation (8) is the key equation to calibrate the sensor, and from calibration measurements, the eight coefficients $A_{\mu }$ are estimated, and then the eight coefficients $S_{\nu }$ are computed. Physically, $S_{0}=\lambda_{0}$ is the Bragg wavelength of the center of cube, $S_{t}$, $S_{h}$, and $S_{\varepsilon }$ are the temperature, humidity, and strain sensitivities, respectively. The cross-sensitivities between two variables are expressed by $S_{th}$, $S_{t\varepsilon }$, and $S_{h\varepsilon }$, while the cross-sensitivity between all variables is $S_{th\varepsilon }$. With the eight coefficients, the response for any point located inside the cube is easily computed by relation (9) and compared with measurement to check the validity of the first-order model with interaction. Then if the validity is correct, the model can predict all the values inside the cube; this is a kind of interpolation. Even if relation (9) gives values for points outside the cube, this kind of extrapolation should not be used because there is no guarantee that the first-order model with interaction is also valid in this extended range. The only way to extend the range is to make eight new measurements in a bigger cube and test the model with control points.

In Equation (8), there are 8 unknowns, i.e., the 8 coefficients $A_{\mu }$ of the two-level factorial design (FD), that should be computed from at least 8 independent measurements. With the choice of the trials ($y_{1}$ to $y_{8}$) depicted by Figure 2, Equation (8) always has a solution that is:

(10) $$A=X^{T}\lambda_{Bi}$$

where X is the design matrix, *T* the transpose operation, A the column vector of the coefficients, and $\lambda_{Bi}$ the column vector of the Bragg wavelengths measured at the vertexes of the cube [27,28,29].

It is worth noting that, based on literature results, we assume a linear behavior with temperature, strain, and humidity [1,2,4,13] for the ranges of variables encountered in normal environmental conditions. This assumption is correct for small temperature and strain ranges, but should always be checked experimentally. This is the goal of the control point ($y_{c}$) located at the center of the cube. If the measurement of this point matches with the first-order model with interaction computed from the eight experimental points ($y_{1}$ to $y_{8}$), then this model is sufficient. If it is not the case, a second-order model with interaction should be tested. That would be the case for the temperature, as it is known that the relation between the Bragg wavelength and the temperature becomes quadratic [32] in an extended temperature range. In that case, it is easy to extend the first-order model with interaction to a second-order model with interaction by including at least the quadratic term $A_{tt}X_{t}^{2}$ in relation (8) and even the other quadratic term $A_{\varepsilon \varepsilon }X_{\varepsilon }^{2}$ and $A_{hh}X_{h}^{2}$ if necessary. Of course, in that case, more than eight experimental points should be measured to estimate the supplementary coefficients $a_{ii}$. Then the optimal experimental design is no longer a cube but can be a composite design, a Box–Behnken design, or a Doehlert design [27].

## 4. Experimental Setup and Results

The experimental setup is the same as the one used in [12]. We need to control three variables: temperature, humidity, and strain. Therefore, the experiment was performed in a climate chamber from WEISS TECHNIK-SB$22^{300}$ that is used to control temperature and humidity, and two different calibrated weights were used to apply the strain on the grating according to:

(11) $$\varepsilon =\frac{mg}{E_{\mathrm{silica}}A_{\mathrm{silica}}+E_{\mathrm{PI}}A_{\mathrm{PI}}}$$

where *m* is the mass of the calibrated weights, *g* is the gravity acceleration (9.81 m/$s^{2}$), $E_{\mathrm{silica}}$ (72 GPa, [33]) and $E_{\mathrm{PI}}$ (2.5 GPa, [34]) are the Young’s modulus of the silica and polyimide, respectively, and finally, $A_{\mathrm{silica}}$ and $A_{\mathrm{PI}}$ are the cross-sections of the fiber with an outer diameter of 125 μm and the polyimide with a thickness around 25 μm, respectively. Then $E_{\mathrm{PI}}A_{\mathrm{PI}}\ll E_{\mathrm{silica}}A_{\mathrm{silica}}$, so that the values of ε are computed for the silica only.

The levels of the variables were set according to Table 1, for testing the humidity sensor in the usual conditions. A Bragg-meter from FiberSensing (FS22) was used to extract the eight spectra corresponding to the eight vertexes $y_{1}$ to $y_{8}$. Then, a Python program was used to extract the eight Bragg wavelengths $\lambda_{Bi}$ with a chosen peak search algorithm, from which the sensitivities $A_{\mu }$ and $S_{\nu }$ were computed.

The level of the factor $y_{c}$ for the control point is shown in Table 1 as well. As it can be seen, the control point is not exactly at the center of the cube, but corresponds to $\Delta T=-0.8{}^{{}^{\circ}}C$ and Δϵ= −1.018 μϵ. Moreover, the control point was measured 10 times to estimate the dispersion of the interrogator. For all the peak search methods used, the variation was always less than 1 pm.

**Figure 3:**
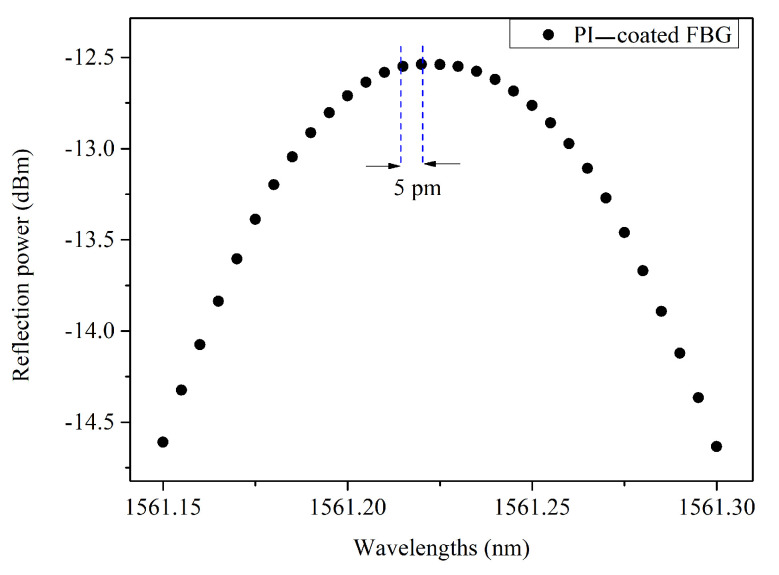
The resolution of the reflection spectrum in our experimental setup is around 5 pm.

A typical example of raw data from the interrogator FS22 is presented in Figure 3. It is worth noting that the FS22 outputs points every 5 pm and can span the wavelength range 1500 nm to 1600 nm with a dynamic range better than 25 dB.

**Figure 4:**
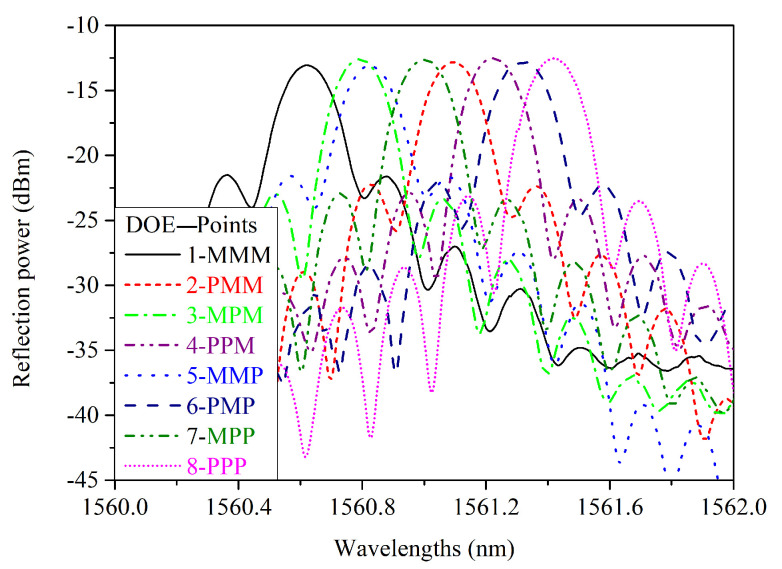
Reflection spectra of PI-FBG for any combination of three factors in two levels (M represents a low level (−1), and P a high level (+1)).

The reflection spectra of the PI-coated FBG for the eight measuring points are presented in Figure 4 where labeling is as follows: low-level (−1) is M, and high-level (+1) is P. Therefore, label 5-MMP, for instance, corresponds to $X_{t}=-1$, $X_{h}=-1$ and $X_{\varepsilon }=+1$ (point $y_{5}$ of Figure 2).

**Figure 5:**
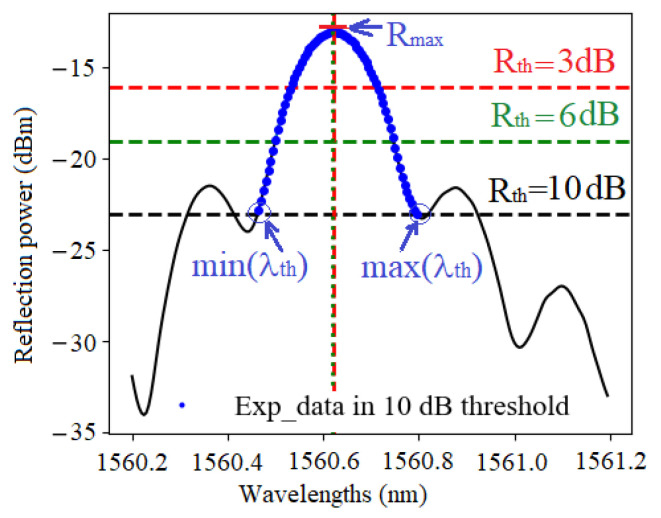
Experimental points with 3, 6 and 10 dB thresholds for the X-dB bandwidth peak detection method.

The reflection spectra clearly exhibit side-lobes, and the main question is how to cope with these side-lobes inside the peak search algorithms that need to work on a selected area around the maximum. To make a fair comparison between the algorithms, we isolated the main lobe in the spectral range of 1560 to 1562 nm, and we selected the data points with the following procedure: we first search for the maximum $R_{\max}$, then we use a threshold of *X*-dB to select the points in the main lobe between $R_{\max}$ and $R_{\mathrm{th}}=R_{\max}-X$. This procedure is displayed in Figure 5 for three thresholds and shows that $\min(\lambda_{th})$ and $\max(\lambda_{th})$ are always the intersections with the main lobe and the threshold.

## 5. Peak-Detection Algorithms

There are many ways to find the Bragg wavelength in a reflection spectrum, and these methods were fully described in the review paper of Tosi [35], where the methods are divided into (1) direct, (2) curve fitting, (3) correlation, (4) transform, and (5) optimization. In the spirit of the design of the experiment approach, we will limit our analysis to the first two categories, as the other ones either require a reference spectrum or need more extensive calculation. It is nevertheless important to note that the calibration method using the factorial design presented here is generic and is thus also perfectly suited for any peak search algorithm.

### 5.1. Maximum

The most common and straightforward method of FBG peak tracking is to detect the maximum value of the reflection spectrum, $R_{\max}$, and then find the corresponding wavelength $\lambda_{\max}$ that is assigned to the Bragg wavelength $\lambda_{B}$. When the interrogator is configured in this mode, the interrogator outputs a file with a series of couples ($R_{\max},\lambda_{\max})$ corresponding to the successive peaks in the spectrum.

From this file, or by applying a maximum search on the raw data, the $S_{\nu }$ coefficients are computed from Equation (8), and the results are presented in Table 2.

It is important to test if the first-order model with interaction is a good assumption. Therefore, Table 2 also gives the value of $\lambda_{cp}$ calculated from the model ($S_{\nu }$ coefficients), and the experimental value $\lambda_{meas}$ measured for $y_{c}$ by using the same peak search algorithm. We clearly see that the agreement is within 1 pm, and there is thus no need to try a second-order model with interaction.

The temperature, humidity and strain sensitivities of PI-coated FBGs are therefore equal to 11.28 pm/${}^{{}^{\circ}}C$, 3.66 pm/%RH and 1.16 pm/μϵ, respectively, whereas cross-sensitivities are quite small. It is interesting to note that the temperature and strain sensitivities matcg well with the values found in the literature for silica-based FBG [2,3]. Moreover, the humidity sensitivity also matches with the results of ([13], Figure 2) for a polyimide thickness around 25 to 30 μm.

### 5.2. X-dB Bandwidth

The principle is depicted in Figure 5. It consists of (1) searching for $R_{\max}$, (2) finding the two intersections $\min(\lambda_{th})$ and $\max(\lambda_{th})$ of the line $R_{\mathrm{th}}=R_{\max}-X$ with the main lobe of spectrum trace, and (3) estimating $\lambda_{B}$ by the midway point between these two intersections:

(12) $$\lambda_{B}=\min\left(\lambda_{th}\right)+\frac{\max\left(\lambda_{th}\right)-\min\left(\lambda_{th}\right)}{2}$$

The results of this procedure for X=1,3,6 and 10 dB are presented in Table 3.

The coefficient units are identical to the units in Table 2. The main fact is the change of sign of the $S_{t\varepsilon }$ coefficient for 6 and 10 dB bandwidths. The control point also confirms the choice of the first-order model with interaction.

### 5.3. Centroid Method or Center of Mass

The centroid algorithm finds the center of the data points as described in relation (13):

(13) $$\lambda_{B}=\frac{\Sigma \lambda_{i}R_{i}}{\Sigma R_{i}}$$

where $R_{i}$ are converted into a linear scale. To apply this method in a reproducible way, we limit the number of points to the ones found by the *X*-dB procedure depicted in Figure 5. The results with X=1,3,6 and 10 dB thresholds are presented in Table 4.

Again, the sign and magnitude of the $S_{t\varepsilon }$ coefficient have changed for 6 and 10 dB bandwidths.

### 5.4. Polynomial Fit

**Figure 6:**
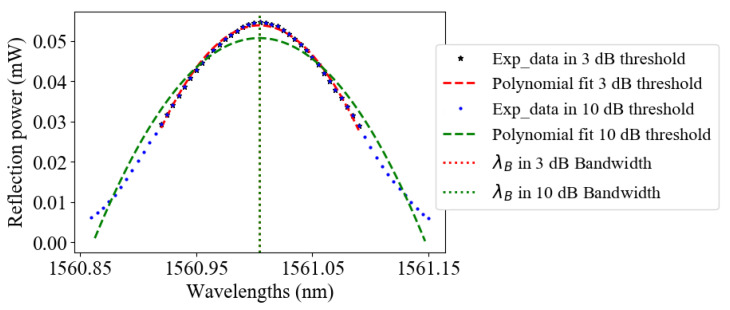
A second-order polynomial fit for linear scaled data points in 3 and 10 dB thresholds.

A second-order polynomial function is used to fit in the main lobe, around $R_{\max}$, the measured FBG reflection spectrum in linear scale (Figure 6), according to relation (14):

(14) $$R\left(\lambda \right)=a_{2}\lambda^{2}+a_{1}\lambda +a_{0}$$

where $a_{1}$ and $a_{2}$ are the coefficients from which the Bragg wavelength is determined as:

(15) $$\lambda_{B}=-\frac{a_{1}}{2a_{2}}$$

Table 5 displays the results for this fit when the number of points is selected by the *X*-dB procedure.

### 5.5. Gaussian Fit

**Figure 7:**
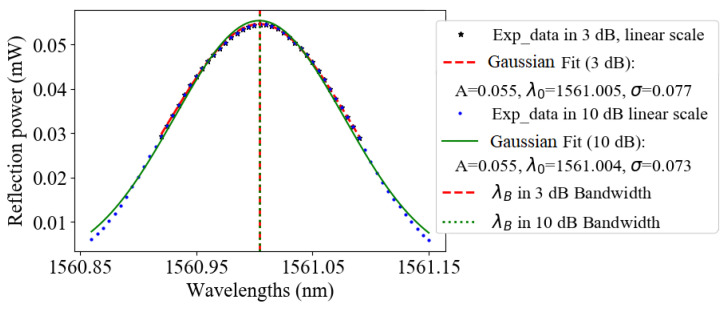
Gaussian fit example for data points on linear scaled data points in 3 and 10 dB thresholds.

The reflection spectrum (in linear scale) around $R_{\max}$ in the main lobe is interpolated with a Gaussian function:

(16) $$R\left(\lambda \right)=Ae^{-\frac{{\left(\lambda -\lambda_{0}\right)}^{2}}{2\sigma^{2}}}$$

where *A* is a multiplicative constant, $\lambda_{0}$ the mean, and σ the standard deviation of the data used in the fitting procedure, as shown in Figure 7. As for the previous case, the number of points used is selected by the *X*-dB procedure whose results are presented in Table 6.

Here, all the thresholds give similar results; there is no sign change, but $S_{t\varepsilon }$ is decreasing with the threshold increase.

### 5.6. Cubic-Spline Fit

**Figure 8:**
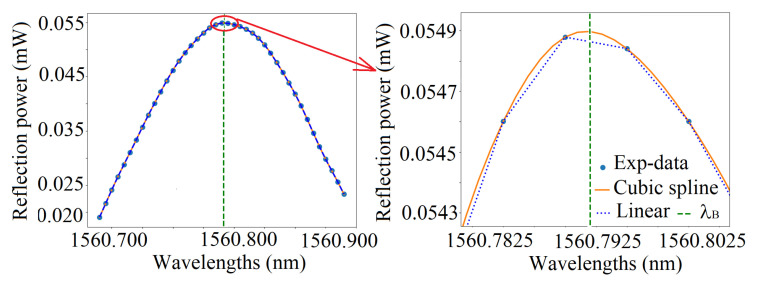
An example of cubic-spline on the experimental data.

The cubic-spline is an interpolation method that uses piece-wise third-order polynomials with continuity up to the second derivative at the measuring points. It is extensively used in many practical applications, and detailed information can be found in [36]. Figure 8 gives an example of such computation, and the Bragg wavelength is computed from the maximum of the spline polynomial around the maximum experimental value of the spectrum. It is clear that the Bragg wavelength will be threshold-independent.

The coefficients of the first order model with interaction calculated from the cubic-spline are displayed in Table 7. The Bragg wavelength of the control point ($\lambda_{cp}$) and measured point ($\lambda_{meas}$) are again in very good agreement with this peak detection method.

## 6. Discussion

**Figure 9:**
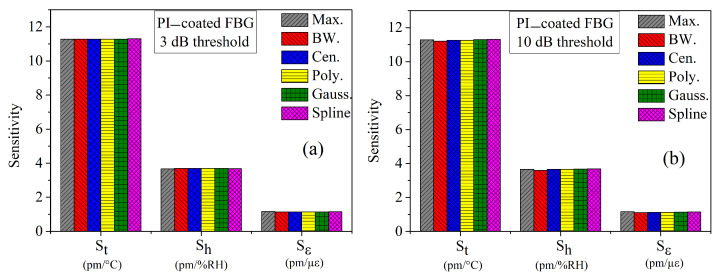
Comparison of main sensitivities of temperature, humidity, and strain, calculated by different peak tracking methods for PI-coated FBG for thresholds of (**a**) 3 and (**b**) 10 dB.

**Figure 10:**
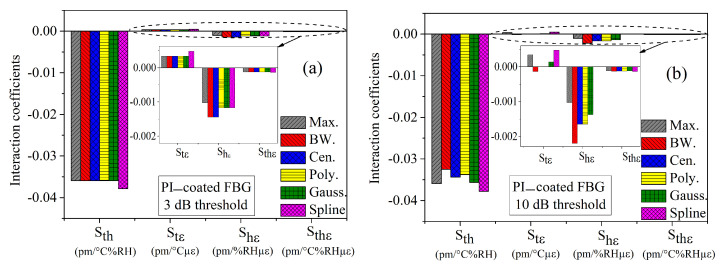
Comparison of interaction coefficients of temperature, humidity, and strain, calculated by different peak tracking methods for PI-coated FBG for thresholds of (**a**) 1 and (**b**) 3 dB.

From the peak detection algorithms of the previous sections, we have calculated the sensitivity coefficients $S_{i}$, as well as the cross-sensitivity coefficients $S_{ij}$ and $S_{ijk}$, as shown in Table 2, Table 3, Table 4, Table 5, Table 6 and Table 7. In this section, we summarize all the results and compare these algorithms together. Figure 9 and Figure 10 present the main and the cross-interaction sensitivities calculated by the six methods for (a) 3 and (b) 10 dB thresholds, respectively.

Rapid inspection of the figures reveals that the main sensitivities are the same for any peak search methods, whereas the cross-sensitivities are quite sensitive to the peak search algorithm. Moreover, dispersion of the results is smaller for the 3 dB threshold. To better highlight the differences between the peak search algorithms, we calculated the mean (μ) and standard deviation (σ) values over the six methods of each sensitivity, as shown in Table 8 and Table 9, for the 1, 3, 6 and 10 dB thresholds.

There is a slight difference in the sensitivities for different thresholds: while the mean values do not differ significantly, the standard deviations are the smallest for the 3 dB threshold. The same conclusion can be drawn for the cross-sensitivity coefficients. Moreover, for the 10 dB threshold, the temperature-strain $S_{t\varepsilon }$ cross-sensitivity becomes very small with a large standard deviation.

The theoretical shape of the reflection spectrum of a perfect FBG is made of hyperbolic sine functions, therefore Gaussian fit and second-order polynomial fit are only approximations. If the window used to select the points in the main lobe is too wide, the fitting quality can decrease. This effect is mainly visible in Figure 6 for the polynomial fit.

There is perhaps a small asymmetry on the whole experimental spectra, but in our computation, we only used the main lobe, which is much more symmetrical. Indeed, the main lobe asymmetry is not visible on the figures, and difficult to quantify, as there is no easy way to obtain a measure of the asymmetry. However, as all the spectra are treated in the same way, we expect a negligible effect on the coefficient computation.

Another interesting comparison point is the computation time for the different peak search algorithms. This is especially important for real-time monitoring. We used Python programming on 2.3 GHz Dual-Core Intel Core i5, with 8 GB of RAM with state-of-the-art numerical tools from Numpy and Scipy modules. Relative times to the 3 dB threshold bandwidth technique with an execution time around 20 μs for the data of Figure 4) are presented in Table 10.

For a given threshold, the fastest method is always the *X*-dB bandwidth and the slowest is always the Gaussian fit. The polynomial fit and the centroid are still relatively fast compared to the Gaussian fit, whereas the spline is threshold independent. When the threshold increases from 3 to 10 dB, the number of data points used in the computation also increases, and all methods except the cubic spline need more time to execute, with a ratio from 1.1 for the X-dB bandwidth method to 1.4 for the Gaussian fit.

Based on our experimental data, the most efficient technique to calibrate the humidity sensor is thus the 3-dB bandwidth, leading to:

(17) $$\lambda_{B}=\left(S_{0}+S_{t}\Delta T+S_{\varepsilon }\Delta \varepsilon +S_{t\varepsilon }\Delta T\Delta \varepsilon \right)+\left(S_{h}+S_{th}\Delta T+S_{h\varepsilon }\Delta \varepsilon \right)\Delta H=S_{0}^{\prime }+S_{h}^{\prime }\Delta H$$

where $S_{0}^{\prime }$ and $S_{h}^{\prime }$ are the offset and effective humidity sensitivity, respectively.

**Figure 11:**
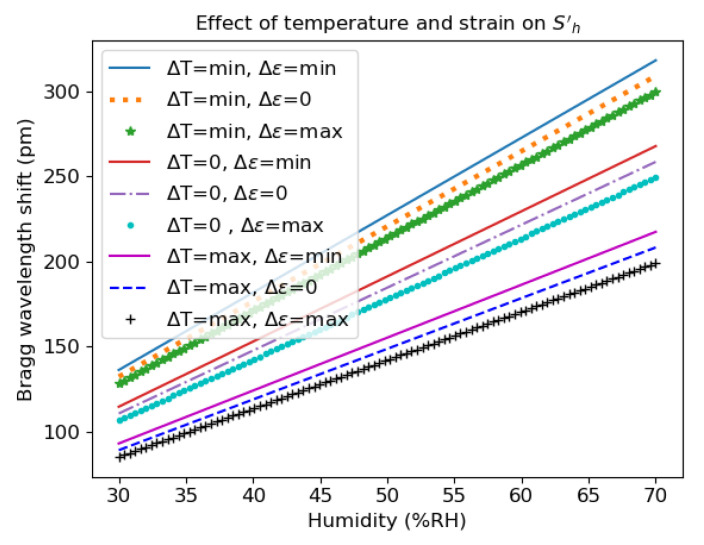
Effective humidity sensitivity for different temperatures and strains.

Table 11 and Figure 11 clearly demonstrate the importance of the interaction terms in the calibration procedure. Indeed, the effective humidity sensitivity ranges from 2.844 pm/%RH to 4.544 pm/%RH. It is also clear that the main parasitic effect is the temperature, i.e., for a given temperature, the lines for the different values of Δε are nearly parallel and in groups of three in Figure 11. It is also important to note that there is an offset that is strongly temperature-dependent.

## 7. Conclusions

The calibration of FBG sensors is vital to making good measurements. This is especially true when the sensor is sensitive to multiple parameters, as the cross-sensitivities affect the effective sensitivity of the main variables. To conduct this calibration in an efficient way, a factorial design with a first-order model with interaction is first tested, and if the control point agrees with the model, the procedure stops there. Otherwise, new calibration points are added to test a second-order model with interaction.

To illustrate this generic procedure, we designed an FBG-based humidity sensor and made a calibration, taking into account the parasitic effects of temperature and strain. We used a three-variable two-level design of the experiment setup and six traditional peak tracking algorithms (maximum, *X*-dB bandwidth, centroid, Gaussian fit, second-order polynomial fit, and cubic spline) to estimate the sensitivities to humidity, temperature and strain, as well as the cross-sensitivities between the variables. To make a fair comparison, the centroid, Gaussian fit and polynomial fit were computed on the same number of points as the one obtained from the *X*-dB threshold criteria. We tested 1, 3, 6 and 10 dB thresholds, and, for this sensor, the 3 dB threshold selection mechanism provided the best results with the smallest dispersion and with always the same sign for the interactions. Amongst the six equivalent methods, the 3 dB bandwidth and second-order polynomial fit with 3 dB threshold are the best suited for fast measurements. After calibration, corrections for temperature, and if needed for strain, are easy to handle. Experimental results also reveal that cross-sensitivity corrections are not negligible, especially for temperature. 

## Figures and Tables

**Table 1:** High and low levels of the factors.

Factor	High Level	Low Level	Center	Control-Point (CP)
Temperature (T)	65$\;{}^{{}^{\circ}}C$	25$\;{}^{{}^{\circ}}C$	45$\;{}^{{}^{\circ}}C$	44.2 $\;{}^{{}^{\circ}}C$
Humidity (H)	70%RH	30%RH	50%RH	50%RH
Strain (ε)	226.65 μ ϵ	44.45 μ ϵ	135.55 μ ϵ	134.532 μ ϵ

**Table 2:** Factorial design experiment results; main sensitivities: $S_{t}$, $S_{h}$, $S_{\varepsilon }$, and cross interaction coefficients: $S_{th}$, $S_{t\varepsilon }$, $S_{h\varepsilon }$, and $S_{th\varepsilon }$ of PI-coated FBG with maximum reflection detection method.

PI-Coated FBG, Maximum		
Coeff.	Coeff. Unit	PI-Coated FBG
$S_{0}$	nm	1561.036
$S_{t}$	$pm{/}^{{}^{\circ}}C$	11.281
$S_{h}$	pm/%RH	3.656
$S_{\varepsilon }$	pm/μϵ	1.159
$S_{th}$	$pm{/}^{{}^{\circ}}C/\%\mathrm{RH}$	$-3.594\times 10^{-2}$
$S_{t\varepsilon }$	$pm{/}^{{}^{\circ}}C/\mu \epsilon$	$+3.430\times 10^{-4}$
$S_{h\varepsilon }$	pm/%RH/μϵ	$-1.029\times 10^{-3}$
$S_{th\varepsilon }$	$pm{/}^{{}^{\circ}}C/\%\mathrm{RH}/\mu \epsilon$	$-1.201\times 10^{-4}$
$\lambda_{cp}$	nm	1561.026
$\lambda_{meas}$	nm	1561.025

**Table 3:** Main and cross sensitivities of PI-coated FBG, calculated using factorial design in X-dB bandwidth algorithms.

PI-Coated FBG, X-dB Method				
**Coeff.**	**1 dB Bandwidth**	**3 dB Bandwidth**	**6 dB Bandwidth**	**10 dB Bandwidth**
$S_{0}$	1561.037	1561.037	1561.037	1561.039
$S_{t}$	11.300	11.281	11.263	11.200
$S_{h}$	3.700	3.694	3.712	3.588
$S_{\varepsilon }$	1.142	1.140	1.131	1.114
$S_{th}$	$-3.687\times 10^{-2}$	$-3.594\times 10^{-2}$	−3.500×10−2	$-3.250\times 10^{-2}$
$S_{t\varepsilon }$	$+1.372\times 10^{-4}$	$+3.430\times 10^{-4}$	$-1.372\times 10^{-4}$	$-1.373\times 10^{-4}$
$S_{h\varepsilon }$	$-1.510\times 10^{-3}$	$-1.441\times 10^{-3}$	$-1.510\times 10^{-3}$	$-2.195\times 10^{-3}$
$S_{th\varepsilon }$	$-1.098\times 10^{-4}$	$-1.201\times 10^{-4}$	$-1.235\times 10^{-4}$	$-1.304\times 10^{-4}$
$\lambda_{cp}$	1561.027	1561.027	1561.027	1561.029
$\lambda_{meas}$	1561.025	1561.025	1561.025	1561.025

**Table 4:** Main and cross sensitivities of PI-coated FBG, calculated using factorial design in a centroid detecting algorithm within two thresholds.

PI-Coated FBG, Centroid Method				
**Coeff.**	**1 dB Threshold**	**3 dB Threshold**	**6 dB Threshold**	**10 dB Threshold**
$S_{0}$	1561.037	1561.037	1561.037	1561.038
$S_{t}$	11.300	11.281	11.263	11.263
$S_{h}$	3.700	3.694	3.713	3.663
$S_{\varepsilon }$	1.142	1.140	1.131	1.131
$S_{th}$	$-3.687\times 10^{-2}$	$-3.594\times 10^{-2}$	$-3.500\times 10^{-2}$	$-3.437\times 10^{-2}$
$S_{t\varepsilon }$	$+1.372\times 10^{-4}$	$+3.430\times 10^{-4}$	$-1.372\times 10^{-4}$	$-3.120\times 10^{-14}$
$S_{h\varepsilon }$	$-1.509\times 10^{-3}$	$-1.441\times 10^{-3}$	$-1.509\times 10^{-3}$	$-1.646\times 10^{-3}$
$S_{th\varepsilon }$	$-1.098\times 10^{-4}$	$-1.201\times 10^{-4}$	$-1.235\times 10^{-4}$	$-1.124\times 10^{-4}$
$\lambda_{cp}$	1561.027	1561.027	1561.027	1561.028
$\lambda_{meas}$	1561.025	1561.025	1561.024	1561.023

**Table 5:** Main and interaction sensitivities of PI-coated FBG, calculated using factorial design using polynomial algorithms.

PI-Coated FBG, Polynomial Fit				
**Coeff.**	**1 dB Threshold**	**3 dB Threshold**	**6 dB Threshold**	**10 dB Threshold**
$S_{0}$	1561.037	1561.037	1561.037	1561.038
$S_{t}$	11.281	11.281	11.288	11.250
$S_{h}$	3.706	3.694	3.688	3.650
$S_{\varepsilon }$	1.140	1.140	1.133	1.131
$S_{th}$	$-3.594\times 10^{-2}$	$-3.594\times 10^{-2}$	$-3.500\times 10^{-2}$	$-3.375\times 10^{-2}$
$S_{t\varepsilon }$	$+4.802\times 10^{-4}$	$+3.430\times 10^{-4}$	$-1.559\times 10^{-14}$	0.000
$S_{h\varepsilon }$	$-1.170\times 10^{-3}$	$-1.166\times 10^{-3}$	$+1.370\times 10^{-3}$	$-1.647\times 10^{-3}$
$S_{th\varepsilon }$	$-1.132\times 10^{-4}$	$-1.201\times 10^{-4}$	$-1.166\times 10^{-4}$	$-1.235\times 10^{-4}$
$\lambda_{cp}$	1561.027	1561.027	1561.027	1561.028
$\lambda_{meas}$	1561.025	1561.025	1561.024	1561.022

**Table 6:** Main and interaction sensitivities of PI-coated FBG, calculated using factorial design using Gaussian fit.

PI-Coated FBG, Gaussian Fit				
**Coeff.**	**1 dB Threshold**	**3 dB Threshold**	**6 dB Threshold**	**10 dB Threshold**
$S_{0}$	1561.037	1561.037	1561.037	1561.037
$S_{t}$	11.281	11.281	11.294	11.288
$S_{h}$	3.706	3.694	3.681	3.675
$S_{\varepsilon }$	1.140	1.140	1.137	1.136
$S_{th}$	$-3.594\times 10^{-2}$	$-3.594\times 10^{-2}$	$-3.531\times 10^{-2}$	$-3.556\times 10^{-2}$
$S_{t\varepsilon }$	$+4.802\times 10^{-4}$	$+3.430\times 10^{-4}$	$+2.058\times 10^{-4}$	$+1.372\times 10^{-4}$
$S_{h\varepsilon }$	$-1.170\times 10^{-3}$	$-1.166\times 10^{-4}$	$-1.300\times 10^{-3}$	$-1.372\times 10^{-3}$
$S_{th\varepsilon }$	$-1.132\times 10^{-4}$	$-1.201\times 10^{-4}$	$-1.132\times 10^{-4}$	$-1.117\times 10^{-4}$
$\lambda_{cp}$	1561.027	1561.027	1561.027	1561.027
$\lambda_{meas}$	1561.025	1561.025	1561.025	1561.025

**Table 7:** Main and interaction sensitivities of PI-coated FBG, calculated using factorial design using cubic-spline fit.

PI-Coated FBG, Cubic-Spline Fit			
**Coeff.**		**Coeff.**	
$S_{0}$	1561.036	$S_{t\varepsilon }$	$+4.802\times 10^{-4}$
$S_{t}$	11.306	$S_{h\varepsilon }$	$-1.166\times 10^{-3}$
$S_{h}$	3.681	$S_{th\varepsilon }$	$-1.138\times 10^{-4}$
$S_{\varepsilon }$	1.154	$\lambda_{cp}$	1561.026
$S_{th}$	$-3.781\times 10^{-2}$	$\lambda_{meas}$	1561.025

**Table 8:** Mean (μ) and standard deviation (σ) over the six peak detection algorithms of sensitivities calculated for 1 and 3 dB thresholds.

	1 dB Threshold		3 dB Threshold	
	μ	σ	μ	σ
$S_{0}$	1561.037	$+7.705\times 10^{-4}$	1561.037	$+6.831\times 10^{-4}$
$S_{t}$	11.292	0.012	11.285	0.010
$S_{h}$	3.692	0.020	3.685	0.015
$S_{\varepsilon }$	1.147	$+8.35\times 10^{-3}$	1.146	$+8.680\times 10^{-3}$
$S_{th}$	$-3.103\times 10^{-2}$	$+1.342\times 10^{-2}$	$+3.625\times 10^{-2}$	$+7.655\times 10^{-4}$
$S_{t\varepsilon }$	$+3.430\times 10^{-4}$	$+1.680\times 10^{-4}$	$+3.659\times 10^{-4}$	$+5.603\times 10^{-5}$
$S_{h\varepsilon }$	$-1.260\times 10^{-3}$	$+2.014\times 10^{-4}$	$-1.230\times 10^{-3}$	$+1.680\times 10^{-4}$
$S_{th\varepsilon }$	$-1.133\times 10^{-4}$	$+3.769\times 10^{-6}$	$-1.224\times 10^{-4}$	$+5.585\times 10^{-6}$

**Table 9:** Mean (μ) and standard deviation (σ) over the six peak detection algorithms of sensitivities calculated for 6 and 10 dB thresholds.

	6 dB Threshold		10 dB Threshold	
	μ	σ	μ	σ
$S_{0}$	1561.037	$+6.325\times 10^{-4}$	1561.037	$+1.49\times 10^{-3}$
$S_{t}$	11.283	0.017	11.265	0.037
$S_{h}$	3.689	0.021	3.653	0.034
$S_{\varepsilon }$	1.141	0.013	1.137	0.016
$S_{th}$	$-3.568\times 10^{-2}$	$+1.11\times 10^{-3}$	$-3.500\times 10^{-2}$	$+1.860\times 10^{-3}$
$S_{t\varepsilon }$	$+9.978\times 10^{-5}$	$+2.803\times 10^{-4}$	$+1.372\times 10^{-4}$	$+2.336\times 10^{-4}$
$S_{h\varepsilon }$	$-8.574\times 10^{-4}$	$+1.110\times 10^{-3}$	$-1.320\times 10^{-3}$	$+7.491\times 10^{-4}$
$S_{th\varepsilon }$	$-1.185\times 10^{-4}$	$+4.612\times 10^{-4}$	$-1.246\times 10^{-4}$	$+6.396\times 10^{-6}$

**Table 10:** Average peak tracking relative time per method.

Method	X-dB	Polynomial	Spline	Centroid	Gaussian
3 dB threshold	1.00	17	27	48	5621
10 dB threshold	1.06	29	27	61	8094

**Table 11:** Humidity sensitivity for different temperature and strain conditions.

Temperature (${}^{{}^{\circ}}C$)	Strain (μϵ)	$S_{h}^{\prime }$(pm/%RH)
25	44.45	4.544
25	135.55	4.413
25	226.65	4.282
45	44.45	3.825
45	135.55	3.693
45	226.65	3.563
65	44.45	3.106
65	135.55	2.975
65	226.65	2.844

## Data Availability

Not applicable.
